# Improving lifestyle obesity treatment during the COVID‐19 pandemic and beyond: New challenges for weight management

**DOI:** 10.1002/osp4.540

**Published:** 2021-07-01

**Authors:** Ann E. Caldwell, Elizabeth A. Thomas, Corey Rynders, Brooke Dorsey Holliman, Cathryn Perreira, Danielle M. Ostendorf, Victoria A. Catenacci

**Affiliations:** ^1^ Department of Medicine Anschutz Health and Wellness Center University of Colorado Anschutz Medical Campus Aurora CO USA; ^2^ Division of Endocrinology Metabolism, and Diabetes Department of Medicine University of Colorado Anschutz Medical Campus Aurora CO USA; ^3^ Rocky Mountain Regional Veterans Affairs Medical Center Aurora CO USA; ^4^ Division of Geriatric Medicine Department of Medicine University of Colorado Anschutz Medical Campus Aurora CO USA; ^5^ Eastern Colorado Veterans Affairs Geriatric Research, Education and Clinical Center Denver CO USA; ^6^ Department of Family Medicine University of Colorado Anschutz Medical Campus Aurora CO USA; ^7^ Adult and Child Consortium for Health Outcomes Research and Delivery Science (ACCORDS) Children's Hospital Colorado University of Colorado Anschutz Medical Campus Aurora CO USA

**Keywords:** Anxiety, behavioral obesity treatment, COVID‐19, home environment, stress

## Abstract

**Objective:**

The COVID‐19 pandemic has resulted in significant changes to daily life and many health‐related behaviors. The objective of this study was to examine how the stay‐at‐home/safer‐at‐home mandates issued in Colorado (March 2020–May 2020) impacted lifestyle behaviors and mental health among individuals with overweight or obesity participating in two separate behavioral weight loss trials (*n* = 82).

**Methods:**

Questionnaires were used to collect qualitative and quantitative data on challenges to weight loss presented by the COVID‐19 pandemic, including changes in dietary intake, physical activity, sedentary behavior, and mental health during the stay‐at‐home/safer‐at‐home mandates.

**Results:**

Using a convergent mixed method approach integrating qualitative and quantitative data, the greatest challenge experienced by participants was increased stress and anxiety, which led to more unhealthy behaviors. The majority perceived it to be harder to adhere to the prescribed diet (81%) and recommended physical activity (68%); however, self‐reported exercise on weekdays increased significantly and 92% of participants lost weight or maintained weight within ±1% 5–6 weeks following the stay‐at‐home mandate.

**Conclusion:**

Study results suggest that obesity treatment programs should consider and attempt to address the burden of stress and anxiety stemming from the COVID‐19 pandemic and other sources due to the negative effects they can have on weight management and associated behaviors.

## 1 INTRODUCTION

Obesity significantly increases the risk for severe COVID‐19 illness, hospitalization, and mortality.^1^, ^2^, ^3^ Thus, the COVID‐19 pandemic has increased the urgency to effectively reduce obesity and related comorbidities (e.g., type 2 diabetes, hypertension) while concurrently presenting substantial challenges to behaviors that facilitate weight management,^4^, ^5^, ^6^ particularly for adults with overweight or obesity.^3^, ^7^, ^8^ It is important to understand how the unprecedented social and institutional efforts to mitigate the spread of the virus have affected diet and physical activity behavior in persons with overweight or obesity, in order to apply insights gained from this collective stress and trauma to inform obesity treatment moving forward.

Within the past year, several recent studies have been published that report on the impact of the COVID‐19 outbreak on adults with overweight or obesity.^3^, ^9^, ^10^, ^11^, ^12^, ^13^, ^14^ The majority of these publications highlight the importance of mental health as well as the challenges in adhering to behavioral recommendations for achieving weight loss. For example, a recent paper examining the effects of the COVID‐19 outbreak on health behaviors among a large international sample (*n* = 7,753) found that individuals with obesity reported higher incidence of weight gain and sharper declines in mental health following the COVID‐19 outbreak compared to respondents with healthy weight or overweight.^9^ In addition, an online survey of 250 people enrolled in health‐related interventions observed high rates of moderate to severe symptoms of anxiety/depression (30%) and symptoms of moderate to severe post‐traumatic stress disorder (68%) that influenced respondents' ability to adhere to behavioral recommendations in their intervention.^10^ Another study surveyed 123 patients with obesity from an obesity medicine clinic and bariatric surgery practice during the stay‐at‐home order, and found that 73% reported increased anxiety, 84% reported increased depression, and 70% reported more difficulty achieving weight loss goals.^11^ Lastly, among participants of an internet‐based behavioral weight loss program in the Northeastern United States, 77% reported experiencing moderate to extreme stress during the stay‐at‐home orders, and stress levels were significantly associated with having less time to spend on weight‐loss efforts.^12^ However, no known study has specifically reported the impact of the COVID‐19 pandemic on individuals with overweight or obesity enrolled in in‐person behavioral weight loss interventions.

The associations between obesity and stress, anxiety, and mental health were recognized prior to the COVID‐19 pandemic.^15^, ^16^ However, the widespread and dramatic increases in stress and anxiety during the pandemic further highlighted the importance of mental health for weight management and present a natural experiment that provides important insights on how to improve behavioral obesity treatment moving forward.

The objective of this study was to examine the acute effects of the COVID‐19 pandemic and stay‐at‐home/safer‐at‐home orders issued in Colorado (26 March 2020/26 April 2020) on adults with overweight or obesity actively participating in an in‐person behavioral weight loss trials to inform potential adaptations that may be necessary to effectively treat obesity in the wake of the COVID‐19 pandemic. While many studies have assessed dietary changes, physical activity and sedentary behaviors and mental health, this study was unique in its use of a convergent mixed methods approach^17^ to integrate qualitative data measuring participants' perceptions of the greatest challenges associated with weight loss and quantitative data measuring changes in mental health, behaviors related to weight loss during the immediate and most strict lock‐down period. The primary hypothesis was that adherence to dietary prescriptions and physical activity recommendations would be adversely affected by the COVID‐19 outbreak and stay‐at‐home orders.

## 2 METHODS

### 2.1 Study design

Participants included 82 adults (80% women) aged 18–55 years with overweight or obesity (BMI 27–45 kg/m^2^) who were actively participating in two separate, ongoing, randomized behavioral weight loss trials at the University of Colorado Anschutz Medical Campus (CU‐AMC): Daily Restriction and Intermittent Fasting Trial‐2 (DRIFT‐2, NCT03411356) and Time Restricted Eating Study (TRE‐Study, NCT03571048). Questionnaires were administered to participants in each trial to measure the immediate effects of the pandemic and stay‐at‐home/safer‐at‐home orders on study participation, including self‐reported changes in diet, physical activity, sedentary behavior, and mental health. Participants in both trials were recruited in cohorts and received similar guidelines‐based^18^ behavioral weight loss programs, including a dietary prescription, goal to increase physical activity, and group‐based behavioral support classes lasting 60–75 min. Classes were shifted from in‐person to video conference on March 24^th^ in DRIFT‐2 and March 18^th^ in TRE‐Study. Both trials were ongoing, thus, comparisons between intervention arms were not conducted.

DRIFT‐2 is a 12‐month interventional trial designed to compare the weight loss efficacy of weekly energy restriction (∼34% from baseline energy requirements) through daily caloric restriction (DCR) or intermittent fasting (IMF; 3 non‐consecutive days/week of 80% energy restriction from baseline requirements). Participants receive a recommendation to increase physical activity gradually up to 300 min/week by month 6, and to maintain this level for the duration of the trial. Group‐based classes take place weekly (months 0–3) and bi‐weekly (months 4–12), with no classes in the follow‐up phase (months 13–18).

The TRE‐Study is a 9‐month pilot study designed to examine the feasibility and acceptability of DCR (∼35% energy deficit from baseline energy requirements) plus time‐restricted eating (TRE; instructions to eat within a 10‐h window starting within 3 h of waking) compared to DCR alone. Participants receive a physical activity recommendation of 150 min/week of moderate intensity physical activity for the full 9‐month intervention. Group‐based classes take place weekly (months 0–3) and monthly (months 4–9).

**FIGURE 1:**
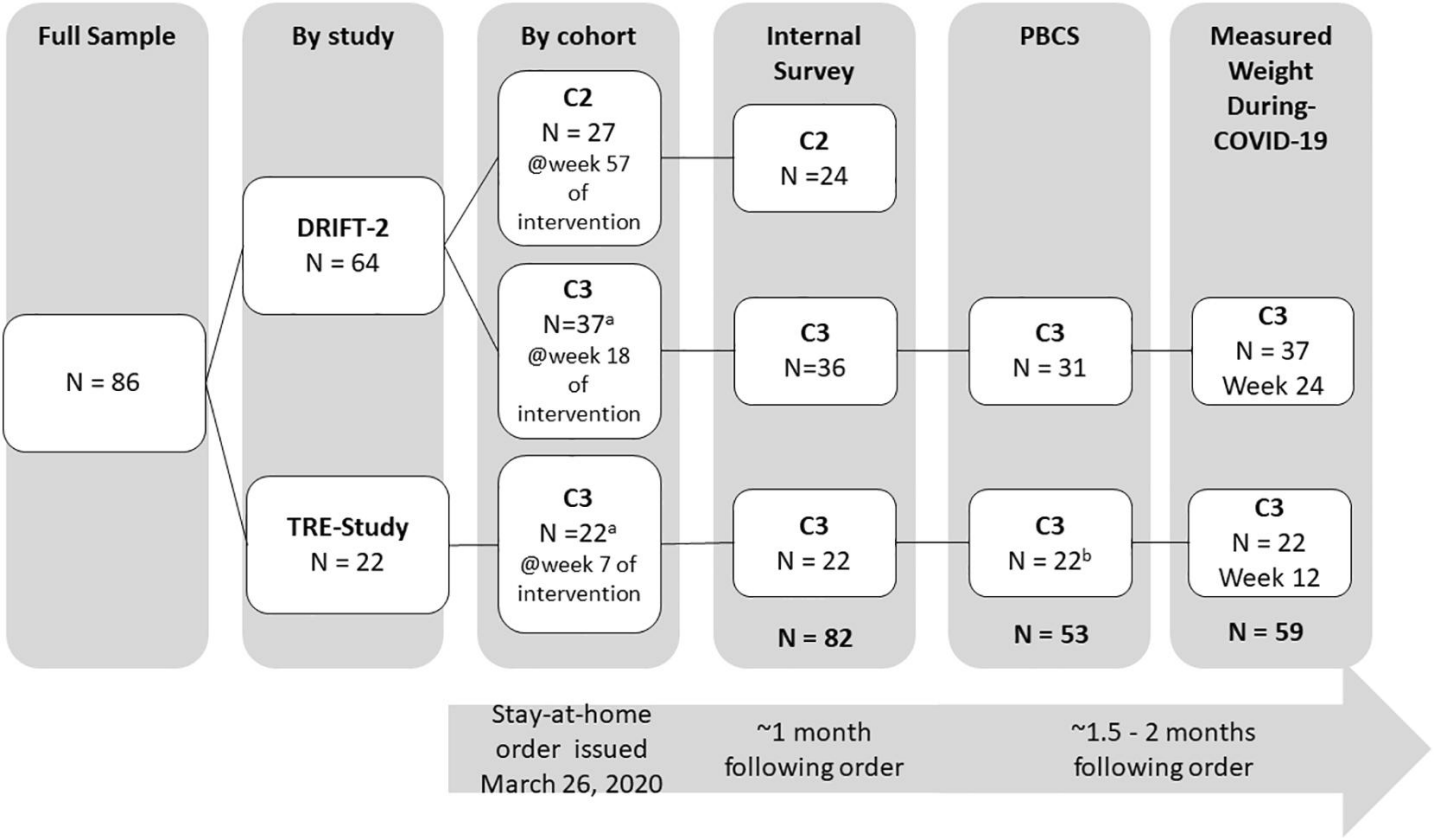
Study design and completion/response rates by study and cohort. DRIFT‐2: Daily restriction and Intermittent Fasting Trial‐2; TRE‐Study: Time Restricted Eating Study, C1: Cohort 1, C2: Cohort 2; C3: Cohort 3; PBCS: Pennington Biomedical COVID‐19 Survey. ^a^Pre‐COVID‐19 weights were measured at the last in‐person class prior to the stay‐at‐home mandate. ^b^Participants in TRE‐Study were given the option to not complete the full generalized anxiety disorder (GAD‐7) measure for “following the COVID‐19 outbreak,” and *n* = 7 chose not to provide responses. Post hoc *t*‐tests or Fischer's exact tests did not reveal significant differences between those who completed the during‐COIVD‐19 GAD‐7 measure and those who chose not to on relevant demographic or baseline characteristics (age, gender, education, race, ethnicity, and BMI) or mental health measures (feeling more sad, stressed, or anxious during COVID‐19, or pre‐COVID‐19 GAD‐7 scores). Participants in DRIFT‐2 were not given the option to not complete this measure and all *N* = 31 respondent provided GAD‐7 responses for “prior to the COVID‐19 outbreak” and “following the COVID‐19 outbreak”

Cohort 1 of DRIFT‐2 and cohorts 1 and 2 of TRE‐Study had already completed the study when the stay‐at‐home order was issued. Cohort 2 of DRIFT‐2 was in the follow‐up phase (intervention week 57), while cohort 3 was attending bi‐weekly classes (week 18) and cohort 3 of TRE‐Study was attending weekly classes (week 7). This analysis was therefore limited to cohorts 2–3 of DRIFT‐2 and cohort 3 of TRE‐Study (Figure 1).

This study was approved by the Colorado Multiple Institutional Review Board at the CU‐AMC.

### 2.2 Survey instruments

An internal survey was developed to measure the immediate impacts of the COVID‐19 pandemic and stay‐at‐home order on study participation using Likert‐style questions and two‐open ended questions to identify challenges to weight loss and study participation posed by the COVID‐19 pandemic (Supplementary Materials 1). It was distributed via Redcap approximately 1 month following the stay‐at‐home order. The Pennington Biomedical COVID‐19 Survey (PBCS) was distributed via Redcap to the two cohorts actively participating in group‐based classes in May 2020 (Cohort 3 of DRIFT‐2, and Cohort 3 of TRE). This publicly available questionnaire measures dietary behaviors, physical activity, sedentary behaviors, mental health, and weight change using a combination of validated questionnaires with minor modifications and investigator‐created questions specific to the pandemic.^19^

#### 2.2.1 Diet behaviors

The internal survey asked participants to rate the extent to which the COVID‐19 pandemic impacted their ability to adhere to the prescribed study diet during the past 30 days on a scale from 1 = *much easier* to 7 = *much harder*. To assess changes in dietary behaviors, the PBCS included a modified Rapid Eating Assessment (REAP‐s)^20^ scale to assess unhealthy dietary patterns. Participants are asked the frequency of engaging in eight unhealthy dietary behaviors during an average week ‘prior to’ and ‘following the pandemic outbreak’ (e.g., breakfast skipping, consuming <2 servings of fruits and vegetables, eating fast food, etc.) on a scale from 1 = *usually/often*, 2 = *sometimes*, 3 = *rarely/never*. Five of the eight items were used to compute scale totals while three questions on skipping breakfast and inadequate intake of fruits/vegetables each day were omitted due to the fasting components of the intervention designs. Scores ranged from 5 to 15, with higher scores indicating healthier diets.

#### 2.2.2 Physical activity and sedentary behavior

The internal questionnaire asked participants to rate the extent to which the COVID‐19 pandemic impacted their ability to adhere to the prescribed physical activity targets during the past 30 days on a scale from 1 = *much easier* to 7 = *much harder*. The PBCS assessed changes in physical activity and sedentary behaviors on weekdays and weekends “prior to” and “following the pandemic outbreak.”

#### 2.2.3 Mental health

The PCBS asked participants to rate the extent to which they generally felt more stressed, anxious, and sad following the COVID‐19 outbreak on a 5‐point Likert scale from 1 = *strongly disagree* to 5 = *strongly agree*, and included the 7‐item generalized anxiety disorder (GAD‐7)^21^ scale. Participants rated anxiety symptoms “prior to” and “following the COVID‐19 outbreak.” Scores ranged from 0 to 21 with higher scores indicating greater anxiety.

### 2.3 Measured weight change

Pre‐COVID‐19 weight was measured by study staff at the last in‐person class prior to the stay‐at‐home order in DRIFT‐2 and TRE‐Study (intervention weeks 18 and 7, respectively). During‐COVID‐19, weights were measured by participants using home scales due to University‐wide restrictions on clinical research. DRIFT‐2 participants used ©BodyTrace smart scales (Palo Alto, CA) that wirelessly transmit weight data to a secure website accessible by researchers 6 weeks following the stay‐at‐home order (intervention week 24). TRE‐Study participants used their own home scales and sent a photograph of the scale to researchers 5 weeks after the stay‐at‐home order was issued (intervention week 12).

### 2.4 Statistical analysis

#### 2.4.1 Quantitative analysis

Baseline demographic and clinical characteristics were summarized using descriptive statistics. Descriptive statistics were also performed for the single‐item survey questions assessing perceptions of study participation, adherence, and mental health following the COVID‐19 outbreak and stay‐at‐home order. To assess changes in anxiety (GAD‐7), unhealthy eating (REAP‐s), and physical activity/sedentary behaviors reported both pre‐ and during‐COVID‐19, Wilcoxon signed‐rank tests were performed due to the non‐normality of score distributions and/or the ordinal scales used. To compare self‐reported versus measured weight change, a paired‐samples *t*‐test was performed on weights prior to and 5–6 weeks after the stay‐at‐home order issuance. Median (interquartile range) and mean (standard deviation) are reported where relevant. In *post‐hoc* analyses, linear regression models were used to determine if anxiety levels during COVID‐19 were associated with participant characteristics, changes in stress or sadness, and weight loss behaviors (Model 1). In Model 2, analyses were adjusted for education and BMI. The alpha level was set at *p* ≤ 0.05.

#### 2.4.2 Qualitative analysis

Content analysis^22^ served as the guiding qualitative methodology for interpretation of the responses to the two open‐ended questions from the internal survey to identify themes describing participants' perceptions of how the COVID‐19 pandemic impacted their weight loss progress and study participation (details in Supplementary Materials 2).

## 3 RESULTS

### 3.1 Participant characteristics

Participant characteristics are summarized in Table 1. Response rates for each measure by study and cohort are detailed in Figure 1.

**TABLE 1:** Demographic and clinical characteristics of the sample

	TRE‐study Mean ± SD *N* (%)	DRIFT‐2 Mean ± SD *N* (%)	Combined Mean ± SD *N* (%)
Total *n*	22	60	82
Age (years)	41.9 ± 8.2	42.3 ± 9.3	42.2 ± 8.9
Baseline weight (kg)	98.1 ± 16.2	96.6 ± 16.4	97.0 ± 16.3
Baseline BMI (kg/m^2^)	34.6 ± 5.3	34.2 ± 4.7	34.3 ± 4.9
Female *N* (%)	18 (72%)	48 (80.3%)	66 (80%)
Ethnicity *N* (%)			
Hispanic or Latino	1 (5%)	17 (22%)	18 (18%)
Not Hispanic or Latino	21 (95%)	59 (78%)	47 (82%)
Race *N* (%)			
Asian	0	5 (8%)	5 (6%)
Black or African American	1 (5%)	5 (8%)	6 (7%)
White	21 (95%)	50 (83%)	71 (87%)
Education *N* (%)			
High School	0	3 (5%)	3 (4%)
Some College	12 (55%)	36 (72%)	48 (59%)
Masters	6 (27%)	11 (18%)	17 (21%)
Doctorate	4 (18%)	10 (16%)	14 (17%)

Abbreviations: BMI, body mass index; DRIFT‐2, Daily Restriction and Intermittent Fasting‐2 study; TRE‐Study, Time Restricted Eating study.

### 3.2 Overall impact of the COVID‐19 outbreak and stay‐at‐home order

Participants were affected by transitioning to working from home (63%), increased childcare responsibilities with school and daycare closures (39%), and increased work hours (26%). A smaller portion of the sample was furloughed or had reduced work hours due to COVID‐19 (16%), and 6% became unemployed. Within the first month following the stay‐at‐home mandate, none of the participants reported COVID‐19 infection, however 4% had a family member sick with COVID‐19, and some had to quarantine themselves (11%) or a family member (5%).

### 3.3 Overall qualitative results on challenges to weight loss progress and study participation

In reviewing all data from both open‐ended questions (122 responses), three primary categories of difficulties related to study participation and achieving weight loss goals became apparent: (1) Mental health, specifically stress and anxiety, (2) dietary intake – specifically access to food and food consumption, and (3) changes in physical activity. A fourth category was noted, in which participants shared comments and observations on how the guidance and requirements associated with study participation influenced their behavior and experience with the COVID‐19 pandemic. Each category is examined more thoroughly below through presentation of both qualitative and quantitative results.

### 3.4 Mental health

#### 3.4.1 Quantitative results

**FIGURE 2:**
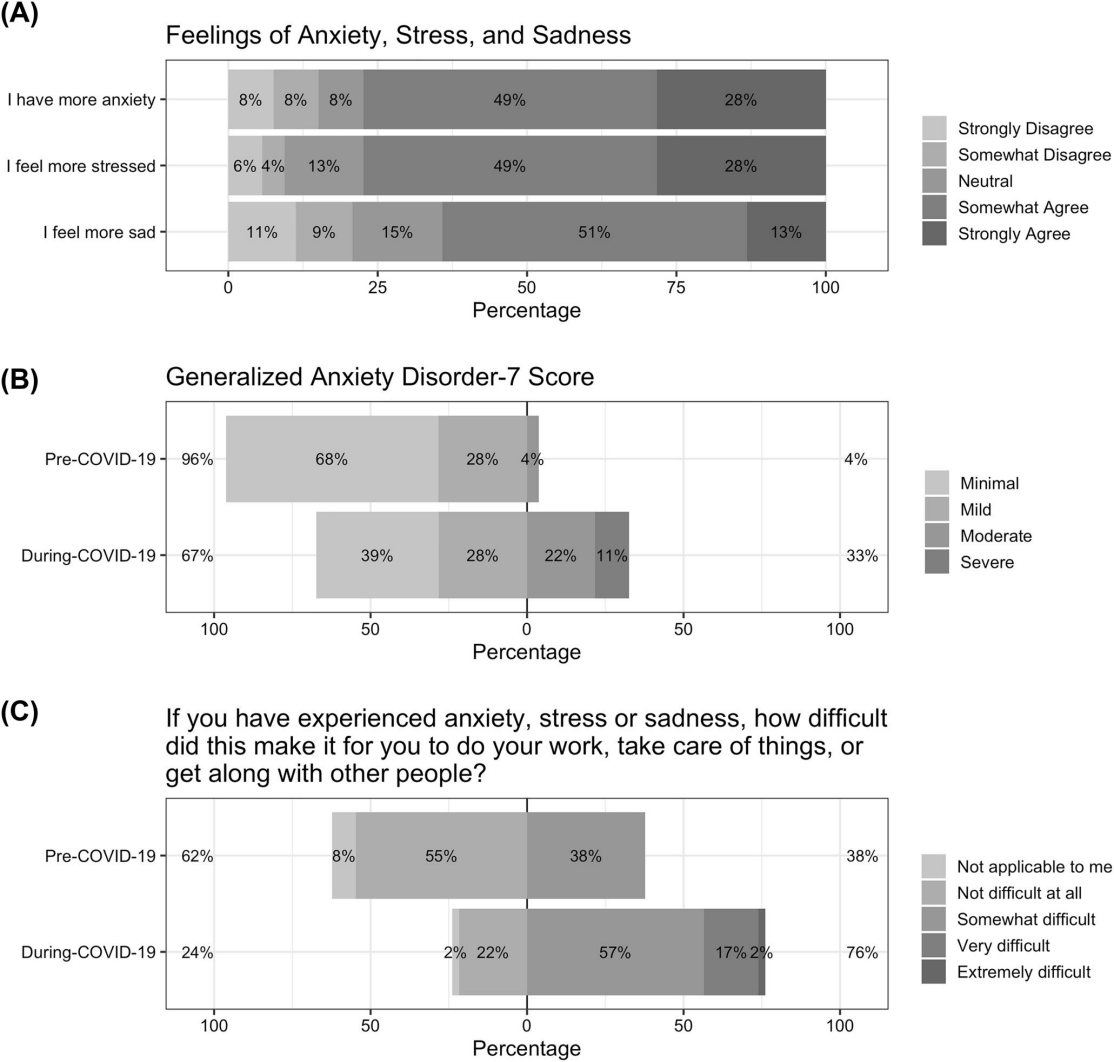
Changes in mental health following the COVID‐19 outbreak from Pennington Biomedical COVID‐19 Survey. The Pennington Biomedical COVID‐19 Survey was completed by the 3^rd^ cohorts of both studies (*n* = 53); however, *n* = 46 participants completed the during‐COVID‐19 Generalized Anxiety Disorder (GAD‐7) measure

Among respondents of the PBCS, the majority either *somewhat* or *strongly agreed* that following the COVID‐19 outbreak they felt more stressed, anxious, or sad (Figure 2). Generalized anxiety scores increased significantly from 3.0 (1.0–6.0) pre‐COVID‐19 to 6.5 (3.0–11.25) during‐COVID‐19, *p* < 0.001. Prior to the COVID‐19 outbreak, no participants met the criteria for high anxiety (GAD score ≥ 15), and 35% reported that their anxiety symptoms made their ability to work, take care of things, or get along with other people *somewhat difficult*. Following the COVID‐19 pandemic, five respondents (11%) met criteria for high anxiety and most (76%) reported difficulty with work, taking care of things, or getting along with people due to anxiety symptoms (Figure 2).

#### 3.4.2 Qualitative results

The greatest challenge to weight loss identified by participants was a significant increase in stress and anxiety as they adjusted to living in pandemic conditions (84 mentions). In addition to a general anxiety regarding COVID‐19 itself, many participants identified the stay‐at‐home order as a root cause of their stress. The stay‐at‐home order greatly impacted participants' daily routines, causing stress and anxiety as they tried to adjust to new circumstances. As one participant described it, “My habits and schedule have been thrown in disarray and my mental state is so overwhelmed by change that I find myself struggling to introduce any more…even in a positive way.” Two of the 'changes' most frequently mentioned as major causes of stress were changes in work and family circumstances. Work stressors included either more or less working hours, anxiety surrounding financial security, and a disruption of established work‐life balance. Family stressors included homeschooling and caring for children, worrying about vulnerable relatives, and tension caused by family members kept in close quarters.

### 3.5 Dietary intake

#### 3.5.1 Quantitative results

**FIGURE 3:**
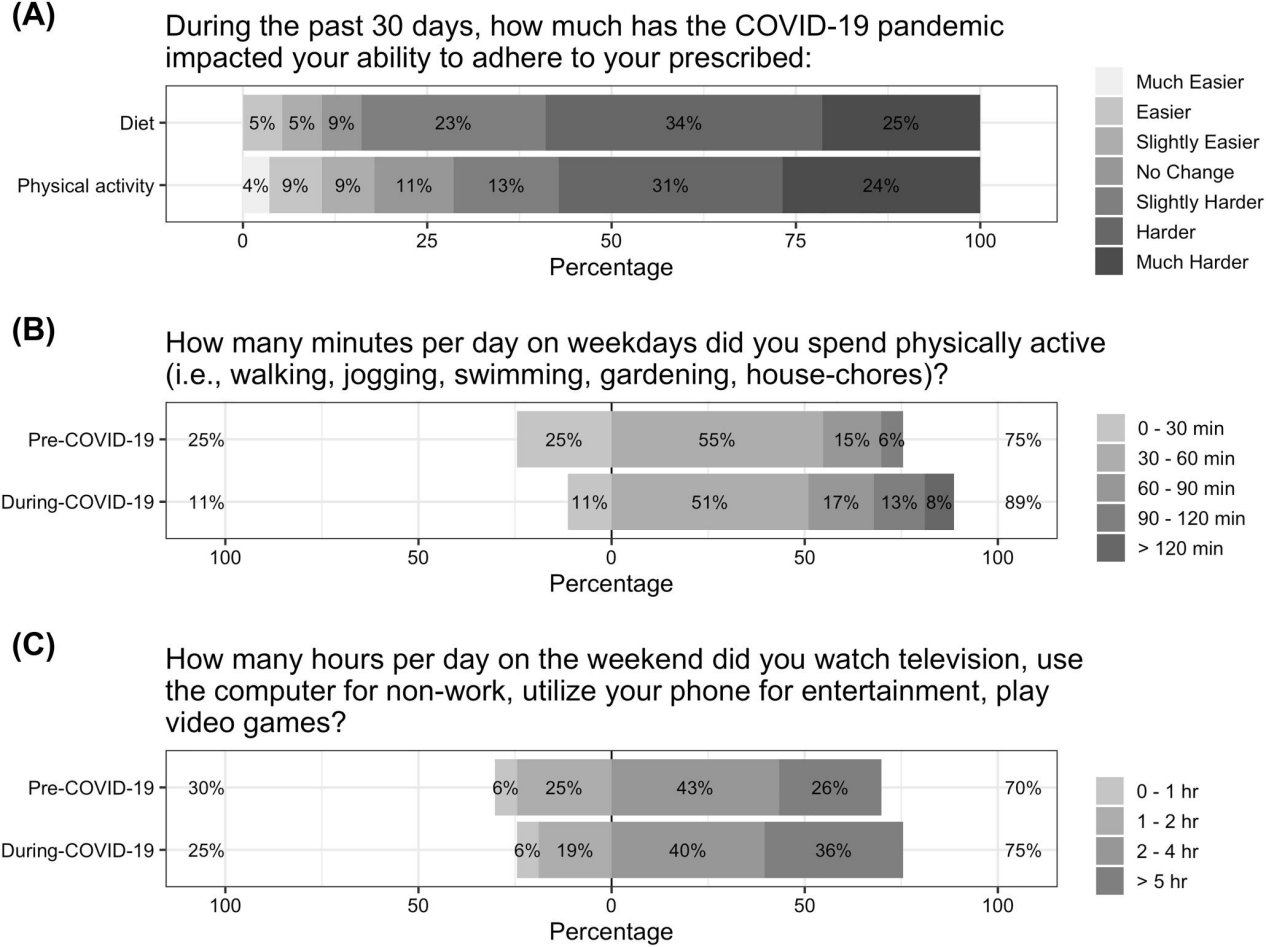
Self‐reported adherence to dietary prescription and physical activity recommendations and changes to weekday exercise and weekend sedentary behavior following the COVID‐19 outbreak. *N* = 80 for the adherence measures, *n* = 53 for physical activity and sedentary behavior. Answer choices for physical activity were ordinal: 0–30, 30–60, 60–90, 90–100 min, and >120 min/day and following Flanagan et al.^9^ were analyzed using the mean value of the given range (i.e., 15, 45, 75, 105, and 135 min respectively) in Wilcoxon signed‐rank tests. Answer choices for sedentary behaviors were ordinal: 0–1, 1–2, 2–4, and ≥5 h/day and were analyzed by using the mean value of the given range (i.e., 30, 90, 180, and 300 min, respectively) in Wilcoxon signed‐rank tests

Among respondents to the internal survey, the overwhelming majority (81%) perceived that it was *harder* to adhere to their prescribed diet following the stay‐at‐home order, while 9% said there was *no change*, and 10% said it was *easier* (Figure 3). Among respondents to the PBCS, 47% perceived that their eating habits were less healthy following the COVID‐19 outbreak, compared to 26% who perceived that their eating habits were healthier. The majority (55%) reported decreases in snacking on fresh fruits and vegetables, and nearly half (45%) perceived increases in snacking on processed foods. REAP‐s unhealthy eating scores increased slightly from 12 pre‐COVID‐19^11^, ^12^, ^13^ to 13 during‐COVID‐19,^11^, ^12^, ^13^, ^14^ though this difference was not significant (*p* = 0.30).

#### 3.5.2 Qualitative results

The second greatest challenge for participants centered on food and food consumption (49 mentions). The primary dietary challenge endorsed by participants was being at home for a longer duration and thus, having constant access to food. Many found the increased stress brought on by being confined at home coupled with this easy access to food led to more snacking and a great deal of stress eating. As one participant put it, “Being home all the time makes me want to eat/cook all the time. [I'm] too quick to use food as soothing for stressful times.” The stay‐at‐home order also meant that participants were unable to visit grocery stores for fresh foods as often as they would like, leading to the consumption of more frozen and shelf goods. “Because I'm limiting trips to the grocery store, I don't always have an abundance of fresh produce and end up eating more dry and frozen items.” Some stores also had limited availability of certain items, making it difficult to follow dietary guidelines.

### 3.6 Physical activity and sedentary behavior

#### 3.6.1 Quantitative results

Among respondents to the internal survey, the majority (68%) perceived that it was *harder* to adhere to their prescribed physical activity following the stay‐at‐home order, while 11% reported *no change*, and 22% said it was *easier* (Figure 3). The time participants self‐reported exercising on weekdays increased significantly from pre‐COVID‐19 to during‐COVID‐19 (*p* < 0.01) which is reflected in a shift in the distribution of responses from pre‐COVID‐19 (IQR: 15–45 min/day) to during‐COVID‐19 (45–75 min/day), though the median remained at 45 min/day (Figure 3). Sedentary behavior on weekends followed a similar pattern, increasing significantly from pre‐COVID‐19 to during‐COVID‐19 (*p* = 0.05), due to a shift in the distribution from pre‐COVID‐19 (90–300 min/day) to during‐COVID‐19 (120–300 min/day), though the median value remained at 150 min/day. Weekend exercise and weekday sedentary behavior did not change significantly from pre‐COVID‐19 to during‐COVID‐19.

#### 3.6.2 Qualitative results

A decrease in exercise and other physical activity presented the third greatest challenge (30 mentions). Primary factors in this challenge were gym closures and an overall decrease in motivation due to increased stress. Gym closures and the cancellation of other activities (yoga, massage, etc.) made it difficult for participants to adhere to their usual exercise routines, especially for those who did not have any equipment at home or those who prefer or need the dynamic of group workouts. In addition, changes in work and familial responsibilities left many participants feeling they had little time and energy to devote to exercise. As one participant reflected, “Balancing work and parenting responsibilities is exhausting and has had a huge impact on my motivation and time to do anything else.” Despite many negative feelings and a lack of motivation, there were a few participants who found the stay‐at‐home order left them with more time to devote to exercise. One participant stated, “Out of boredom I do think I am getting more exercise than I would have in the last month. Many more walks with COVID‐19 than without.”

### 3.7 Study participation and weight change

#### 3.7.1 Quantitative results

**FIGURE 4:**
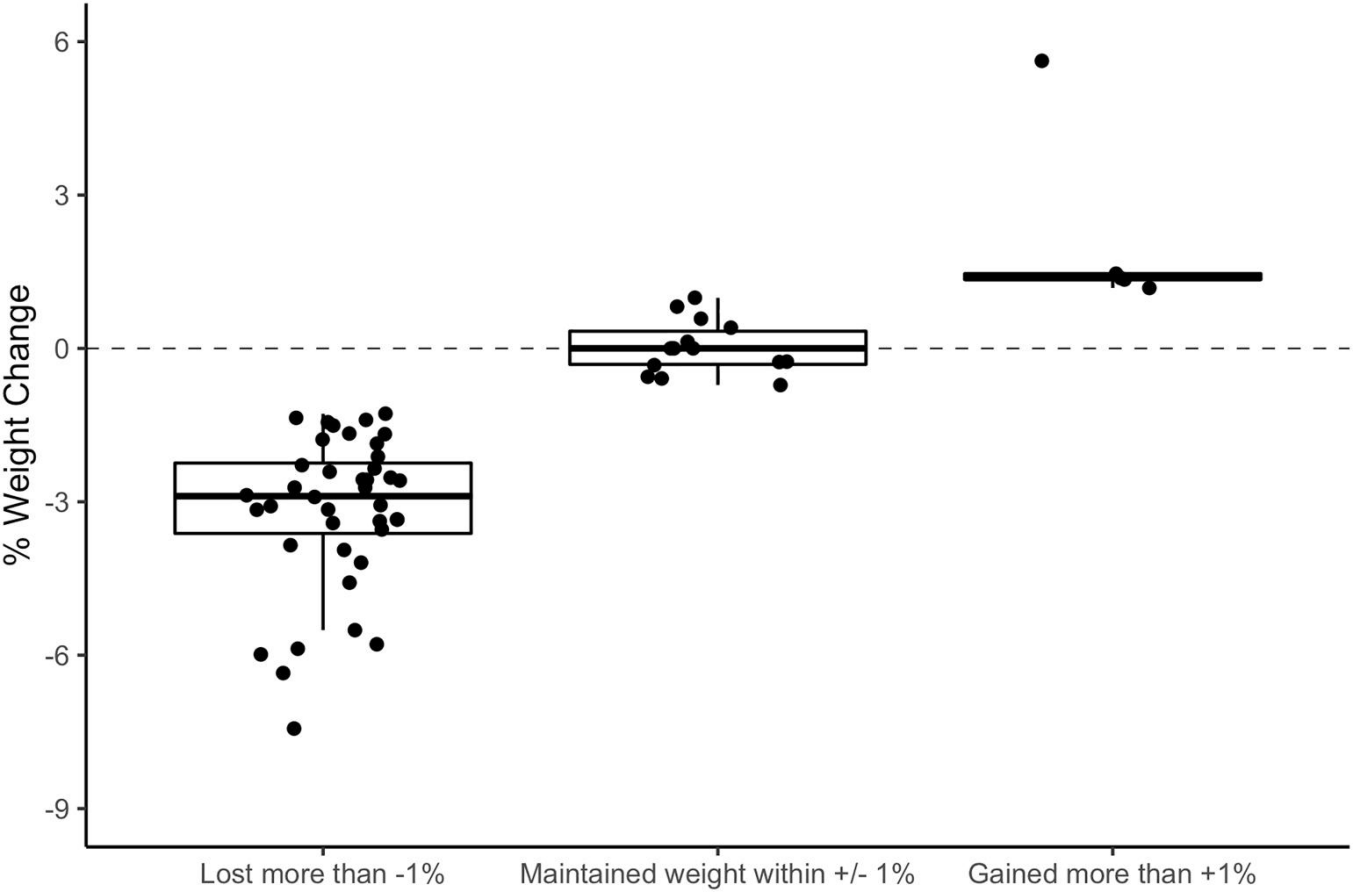
Percent weight change

Half of participants reported that changing classes from in‐person to video conference negatively impacted their enjoyment of classes, while the other half reported enjoying video conference classes *the same* or *more*. Among respondents to the PBCS, 40% perceived that their weight *stayed the same* following the COVID‐19 outbreak, while 25% perceived that they had *lost weight* and 34% perceived they had *gained weight*. However, these perceptions were not supported by the measured weight data which showed that the overwhelming majority lost >1% of their body weight (68%) or maintained weight within ±1% (24%), with just 8% (*n* = 5) gaining >1% body weight during the strictest stay‐at‐home/safer‐at‐home period of the pandemic (Table 2, Figure 4). Mean weight decreased significantly from the last in‐person weight pre‐COVID‐19 (93.66 kg [14.46]) to those measured ∼1.5 months following the stay‐at‐home mandate (91.84 kg [14.53], *p* < 0.001).

**TABLE 2:** Measured weight change

	Start of COVID‐19 (*n* = 59)	During COVID‐19 (*n* = 59)	*p*‐value
Weight (kg)			<0.001^a^
Mean (SD)	93.66 (14.46)	91.84 (14.55)	
Range	61.18–129.55	60.82–129.45	
∆ Weight (kg)			<0.001^a^
Mean (SD)	‐	−1.82 (2.13)	
Range	‐	−6.36–5.00	
∆ Weight (%)			<0.001^a^
Mean (SD)	‐	−1.97 (2.31)	
Range	‐	−7.44–5.62	

^a^ Paired samples *t*‐test. Weight was measured by study staff at the last in‐person class prior to the stay‐at‐home order in DRIFT‐2 and TRE‐Study (intervention week 18 and 7, respectively). During COVID‐19, weights were measured by participants using home scales due to University‐wide restrictions on clinical research. DRIFT‐2 participants used ©BodyTrace smart scales (Palo Alto, CA) that wirelessly transmit weight data to a secure website accessible by researchers 6 weeks following the stay‐at‐home order (intervention week 24). TRE‐Study participants used their own home scales and sent a photograph of the scale to researchers 5 weeks after the stay‐at‐home order was issued (intervention week 12).

#### 3.7.2 Qualitative results

Several participants reported finding participation in the study helpful during this difficult time (14 mentions). They made such statements as “it is helping me to stay on track when otherwise I would have given up,” and “having fast days is one of the few things that is helping me to stay focused.” Most acknowledged that sticking to program guidelines was difficult, and they often failed to follow all steps and restrictions. However, they also stated they continued to see results and hoped to maintain their progress in the future. “Even though I am anxious and stressed I keep trying to remind myself that I've worked so hard to lose weight during this study. I don't want to throw all that hard work away.” Video conference classes were more convenient for many and allowed participants to stay in touch and on track. “I enjoy the virtual meetings because I don't really live close so not having the commute is nice and still getting to see everyone keeps me accountable.”

### 3.8 Association of anxiety with mental health, weight loss behaviors and weight change

Liner regression models were used to examine the relationship between GAD‐7 anxiety levels during the COVID‐19 pandemic with demographic variables, mental health variables, diet, physical activity and sedentary variables, as well as weight change (Table 3). Anxiety levels were significantly and positively associated with BMI and education. Higher anxiety levels were associated with feeling more stressed and more sad following the pandemic outbreak, as well as finding it harder to adhere to the prescribed diet, even after controlling for education and BMI (F [3,39] = 3.93, *p* = 0.02). Higher anxiety levels were also associated with REAP‐s unhealthy eating scores (F [1,44] = 5.44, *p* = 0.02) and difficulty adhering to the physical activity recommendations (F [1,42] = 5.54, *p* = 0.02); though neither association remained significant when controlling for BMI and education. Anxiety levels were not significantly associated with any other weight loss behaviors or weight change. Interestingly, BMI was significantly associated with difficulty adhering to physical activity recommendations (F [3,39] = 4.00, *p* = 0.05) when controlling for anxiety levels and education, but not REAP‐s unhealthy eating scores.

**TABLE 3:** Association between generalized anxiety scores and mental health, weight loss behaviors, weight change, and motivation during COVID‐19 stay‐at‐home mandates

	Model 1: Unadjusted beta (95% CI)	Model 2: Model 1 + BMI + Education beta (95% CI)
Mental health		
Feeling more stressed following COVID‐19 pandemic	0.12*** (0.07, 0.17)	0.12*** (0.06, 0.18)
Feeling more sad following COVID‐19 pandemic	0.09** (0.02, 0.15)	0.12** (0.04, 0.18)
Dietary behaviors		
Rate how much the COVID‐19 pandemic impacted your ability to adhere to prescribed diet^a^	0.10** (0.4, 0.16)	0.08* (0.02, 0.15)
REAP‐s unhealthy eating scores during COVID‐19	−0.13* (−0.25, −0.02)	−0.11 (−0.24, 0.02)
Physical activity and sedentary behaviors		
Rate how much the COVID‐19 pandemic impacted on ability to adhere to the prescribed physical activity^a^	0.12* (0.02, 0.23)	0.06 (−0.05, 0.17)
Weekday exercise minutes during COVID‐19	−0.95 (−2.9, 1.01)	−0.67 (−2.88, 1.53)
Weekend exercise minutes during COVID‐19	−1.36 (−3.63, 0.91)	−0.78 (−3.32, 1.77)
Weekday sedentary time during COVID‐19	0.46 (−4.31, 5.23)	−1.52 (−6.76, 3.72)
Weekend sedentary time during COVID‐19	1.83 (−3.45, 7.11)	−0.14 (−5.99, 5.71)
Weight change		
Kilograms	−0.06 (−0.19, 0.06)	−0.08 (−0.22, 0.06)
%	−0.05 (−0.19, 0.08)	−0.08 (−0.23, 0.08)

Abbreviations: BMI, body mass index; CI, Confidence Intervals; REAP‐s, Rapid Eating Assessment scale.

^a^ *N* = 43.

*p < 0.05, **p < 0.01, and ***p < 0.001.

## 4 DISCUSSION

This study revealed important insights on the specific challenges faced by individuals with overweight or obesity who were actively participating in two separate behavioral weight loss studies during the COVID‐19 outbreak and issuance of stay‐at‐home orders (spring, 2020). In the first month following the stay‐at‐home mandate, qualitative results revealed that participants were most distinctly affected by stress and anxiety, secondarily by challenges associated with the home food environment and reduced access to fresh foods, and to a lesser degree, challenges associated with physical activity and exercise. These challenges were partly mitigated by continuing to participate in the weight loss studies, and only five participants gained >1% of body weight in this timeframe.

The increased level of stress and anxiety affected participants' ability and desire to adhere to the program. Much of this stress was prompted by the stay‐at‐home order, which significantly disrupted daily routines and intensified pressures associated with work and family responsibilities. Declines in mental health were also evident in the quantitative results with increases in perceived stress, anxiety, and sadness, as well as significant increases in generalized anxiety (GAD‐7) scores and an increase in those who expressed difficulty with work and other responsibilities due to anxiety symptoms. While the GAD‐7 scores in this sample were much lower than those observed in a larger, international sample of individuals with obesity using the same measure (pre‐COVID‐19 mean 3.55 vs. 10.43, respectively), the median GAD‐7 scores in the present sample more than doubled in response to the COVID‐19 outbreak, a similar scale of increase observed in individuals with obesity in the larger study.^9^ The lower anxiety scores in the present sample likely reflect screening for major depression and other significant mental health disorders prior to study participation. With the added stress and anxiety came a loss of time and energy, and a corresponding increase in difficulty performing weight loss behaviors.

Dietary intake was more negatively impacted by the COVID‐19 outbreak and stay‐at‐home order than physical activity in both the qualitative and quantitative results. The qualitative analysis revealed that easy access to food at home led many participants to partake in snacking and stress eating. In addition to increased stress and constant access to foods, limited access to healthy foods made adherence to dietary recommendations challenging. Nearly half of respondents perceived *less healthy* eating habits and increases in snacking on processed foods, while the majority perceived decreases in snacking on fresh fruits and vegetables. While 81% perceived more difficulty adhering to the dietary prescriptions, fewer (68%) perceived more difficulty adhering to physical activity recommendations. Closure of fitness facilities and perceived lack of time and energy made adhering to physical activity recommendations more difficult for some study participants. However, several also noted that they were exercising more since the pandemic began, and the distribution of physical activity self‐reported on weekdays increased significantly.

Notwithstanding the many barriers and hardships related to the COVID‐19 pandemic and resultant stay‐at‐home order, several participants found study participation helpful during this time. It helped to provide a sense of structure and accountability. Though nearly all participants experienced negative impacts on their weight‐loss journey due to the COVID‐19 pandemic, most seemed hopeful that they would be able to get back on track once the worst of it was over. Changing the format of classes from in‐person to video conference was perceived as neutral or increased enjoyment for half of the sample, while the other half enjoyed in‐person classes more. This suggests that in‐person group‐based support is valued for many individuals but offering a video conference option will appeal to others.

Despite study participation, 34% of the sample perceived they had gained weight following the stay‐at‐home order, a similar proportion to that observed in a large, international sample of individuals with obesity not participating in a weight loss study.^9^ However, measured weights contradicted this finding, showing that a smaller proportion actually gained weight and among them, the magnitude of weight gain was small. The effects of the pandemic may have slowed the weight loss participants were experiencing prior to the pandemic, leading to higher perceptions of weight gain, but mean weight was significantly reduced over the 5–6 weeks following the stay‐at‐home order, and just 5 participants (8%) experienced weight gain >1%.

Qualitative and quantitative findings from the current study complement those of a recently published study examining stress and weight loss behaviors among adults enrolled in online weight loss program during the same time‐frame.^12^ Pellegrini et al. reported significant associations between perceived stress following the pandemic outbreak with BMI, education, other mental health measures, and less time to dedicate to weight loss efforts. Stress in their sample was also associated with greater difficulty staying on track with eating habits, though this relationship was not significant after adjusting for BMI and education. The current study examined associations between anxiety with weight loss behaviors and weight. In line with the findings of Pellegrini et al., anxiety levels following the pandemic outbreak were significantly associated with BMI and education. Even in the small sample size of the present study, significant associations were observed between anxiety and other measures of mental health (i.e., increased stress and sadness) both before and after adjusting for BMI and education. Anxiety levels were also associated with more difficulty adhering to the dietary and physical activity recommendations of the trials, as well as less healthy eating patterns measured with the REAP‐s scale. However, the association between anxiety and dietary adherence was the only relationship that remained significant after adjusting for BMI and education. In both studies, the associations between stress and anxiety with eating behaviors were more evident than the relationship with stress and anxiety with measures of physical activity. Finally, among this sample of participants of two behavioral weight loss trials, anxiety levels following the pandemic were not associated with weight change.

The convergent mixed‐methods approach integrating qualitative and quantitative data is a strength of this study, as it provides more detailed and nuanced information about the effects of the pandemic on weight loss in this population. Specifically, the analysis of qualitative data clearly showed that stress and anxiety related to the pandemic and stay‐at‐home order presented the biggest challenges with respect to weight loss, with the effects on diet and exercise often stemming from stress and anxiety. In the similar, but exclusively quantitative study of the effects of COVID‐19 on participants of an online weight loss trial,^12^ there was not a significant association observed between stress and eating in response to emotions rather than hunger (i.e., emotional eating). However, the qualitative analysis of open‐ended responses from this study revealed that stress eating was identified as one of the most challenging aspects of working toward weight loss goals following the COVID‐19 pandemic. The former results suggest that emotional eating was not associated with stress during this time‐period, while the latter provides a clear message that stress related eating was a significant difficulty for weight loss for some individuals. Another recent paper of data from 173 adults of any BMI also found that 52% of the sample reported increases in eating in response to stress during COVID‐19 stay‐at‐home safety measures.^23^ Taken together, these nascent lines of evidence suggest that weight loss behavior therapy could be improved by proactively providing stress eating support as a part of the overall curriculum, or as targeted support for those experiencing major life stressors. This support is not currently specifically addressed by the current obesity treatment guidelines^18^, ^24^ outside the general recommendation to identify and problem solve barriers to behavior changes. Providing stress eating support strategies during weight loss interventions may help mitigate some of the well documented weight regain following loss, as most people will experience stressful events throughout their lives.

The insights uncovered by the current study to potentially improve obesity treatment should be interpreted keeping several limitations in mind. The findings are not necessarily generalizable, as it was conducted in a small convenience sample of primarily highly educated, White women participating in a weight loss trial in Colorado. Thus, the perspectives on the impact of the COVID‐19 pandemic may be different than others not attempting to lose weight, across race and ethnic subgroups, or across various regions in the US or around the world. This is particularly important because obesity disproportionately affects racial/ethnic minority groups,^25^, ^26^ and Black and Latino individuals bear a disproportionate burden of COVID‐19 infection and poorer outcomes resulting from COVID‐19.^27^, ^28^ It is well‐recognized that the racial and ethnic groups represented in lifestyle weight loss studies are often not representative of the U.S. population.^29^ However, the present findings on the pervasive impact of the COVID‐19 pandemic on mental health are in line with studies with larger samples,^9^, ^10^ and those in similar sized samples in different regions (12, 23), and those who were not necessarily trying to lose weight.^23^ Nonetheless, future behavioral weight loss trials should include strategies to improve recruitment of individuals from minority groups to better determine the potential public health impact of these interventions in groups that might benefit most from weight loss. In addition, data on pre‐COVID‐19 behaviors and mental health were collected retrospectively. However, the changes in daily life in response to the stay‐at‐home order were both significant and rapid, which should have allowed participants to reflect accurately on changes in their mental health and behaviors over that short period of time. The use of self‐reported rather than objective measures of diet and physical activity can be considered a limitation; however, including objective measures was outside the scope of this study focused on perceptions of the acute effects of the COVID‐19 pandemic and stay‐at‐home order. A follow‐up of these findings with objective behavioral measures collected in each study is planned. Lastly, the use of home scales for during‐COVID‐19 weight measures (rather than clinic weights on a calibrated scale) could have introduced measurement error but was required due to clinical research restrictions.

Our results provide important insights for obesity treatment that are relevant beyond the pandemic. First, ongoing and developing programs for obesity treatment should consider the burden of life stressors and anxiety, and the downstream effects stress and anxiety can have on eating behaviors and weight gain. While the widespread nature of high levels of stress and anxiety resulting from a global pandemic are unique to the current situation, stress and anxiety are commonly encountered throughout life (e.g., major life events like divorce, childbirth, death of family member, move, and job change) and may similarly affect individuals' daily behaviors and bandwidth to focus on weight management and health. The economic recession resulting from the COVID‐19 pandemic is expected to continue for the foreseeable future and increased financial stress will be common for years to come. Among those who worked from home during the stay‐at‐home/safer‐at‐home orders in the present study sample, increased access to food throughout the day presented a major challenge to weight management. It has been estimated that 25%–30% of the workforce will continue to work from home following the pandemic.^30^ Therefore, specific modules on managing stress, stress eating, and how to make the home environment more conducive to healthy eating may need to be emphasized in lifestyle obesity treatment programs during the ongoing COVID‐19 pandemic and beyond.

## CONFLICT OF INTEREST

The authors declared no conflict of interest.

## AUTHOR CONTRIBUTIONS

Ann E. Caldwell, Elizabeth A. Thomas, and Corey Rynders conceived of the analyses. Victoria A. Catenacci, Corey Rynders and Elizabeth A. Thomas conceived of, designed, and obtained funding for the weight loss trials. Ann E. Caldwell, Danielle M. Ostendorf, and Victoria A. Catenacci designed the internal COVID‐19 questionnaire. Ann E. Caldwell and Elizabeth A. Thomas performed quantitative data analysis. The qualitative analysis was conducted by Brooke Dorsey Holliman and Cathryn Perreira under the guidance of Brooke Dorsey Holliman. Ann E. Caldwell, Elizabeth A. Thomas, and Corey Rynders interpreted the quantitative data analysis. Ann E. Caldwell, Elizabeth A. Thomas, and Brooke Dorsey Holliman drafted the manuscript. Ann E. Caldwell, Elizabeth A. Thomas and Corey Rynders generated tables and figures. All authors were involved in writing and revising the manuscript and approved the final version of the manuscript.

## Supporting information

Supplementary Material

Supplementary Material
