# Supplementary material for: Improving lifestyle obesity treatment during the COVID‐19 pandemic and beyond: New challenges for weight management
Source: Obes Sci Pract. 2021 Jul 1;8(1):32–44. doi: 10.1002/osp4.540 (PMC8441901; doi:10.1002/osp4.540)
Supplement: Supplementary file 1 — Supplementary Material [file OSP4-8-32-s002.docx]

**TITLE:** Improving Lifestyle Obesity Treatment During the COVID-19 Pandemic and Beyond: New Challenges for Weight Management

**AUTHORS:** Ann E. Caldwell, PhD^1,2^, Elizabeth A. Thomas MD^1,2,3^, Corey Rynders PhD^4,5^, Brooke Dorsey Holliman, PhD^6,7^, Cathryn Perreira, MA^7^, Danielle M. Ostendorf, PhD^1,2^, Victoria A. Catenacci MD^1,2^

**AFFILIATIONS:**

^1^ Department of Medicine, Anschutz Health and Wellness Center, University of Colorado Anschutz Medical Campus, Aurora, CO, USA

^2^ Division of Endocrinology, Metabolism, and Diabetes, Department of Medicine, University of Colorado Anschutz Medical Campus, Aurora, CO, USA

^3^Rocky Mountain Regional Veterans Affairs Medical Center, Aurora, CO, USA

^4^ Division of Geriatric Medicine, Department of Medicine, University of Colorado Anschutz Medical Campus, Aurora, CO, USA

^5^Eastern Colorado Veterans Affairs Geriatric Research, Education, and Clinical Center, Denver, CO, USA

^6^Department of Family Medicine, University of Colorado Anschutz Medical Campus, Aurora, CO, USA

^7^Adult and Child Consortium for Health Outcomes Research and Delivery Science (ACCORDS), Children’s Hospital Colorado, University of Colorado Anschutz Medical Campus, Aurora, CO, USA

**Supplementary Materials 1.** *Internal Survey*

**1. During the past 30-days did you experience any of the following: (Check all that apply)**

1) became sick with COVID 19

2) was quarantined due to COVID-19

3) worked from home due to COVID-19

4) became unemployed due to COVID-19

5) was furloughed or reduced work hours due to COVID-19

6) experienced increased work because considered an essential service provider due to COVID-19

7) Increased child care responsibilities because schools and daycares were cancelled

8) a family member was sick with COVID-19

9) a family member was quarantined due to COVID-19

**2.** Please rate how much the COVID-19 pandemic has impacted your **ability to adhere to your** **prescribed diet** during the past 30 days. Scores can range from “Much easier” (1) to “Much harder” (7).

| Much easier | Easier | Slightly easier | No change | Slightly harder | Harder | Much harder |
| --- | --- | --- | --- | --- | --- | --- |
| 1 | 2 | 3 | 4 | 5 | 6 | 7 |

**3.** Please rate how much the COVID-19 pandemic has impacted your **ability to adhere to your prescribed physical activity** during the past 30 days. Scores can range from “Much easier” (1) to “Much harder” (7).

| Much easier | Easier | Slightly easier | No change | Slightly harder | Harder | Much harder |
| --- | --- | --- | --- | --- | --- | --- |
| 1 | 2 | 3 | 4 | 5 | 6 | 7 |

**4.** Please rate how much the change to virtual group weight loss classes due to the COVID-19 pandemic has impacted your **enjoyment of group weight loss classes** during the past 30 days. Scores can range from “I like virtual classes much less than in person classes” (1) to “I like virtual classes much more than in person classes” (7). Please choose N/A if you are at a stage of the intervention that no longer has group classes.

| Much less | More | Slightly more | The same | Slightly more | More | Much more | N/A |
| --- | --- | --- | --- | --- | --- | --- | --- |
| 1 | 2 | 3 | 4 | 5 | 6 | 7 |  |

**5.** Please rate how much the COVID-19 pandemic has impacted your **motivation to participate in a weight loss research study** during the past 30 days. Scores can range from “much less motivated” (1) to “much more motivated” (7).

| Much less motivated | Less motivated | Slightly less motivated | No change | Slightly more motivated | More motivated | Much more motivated |
| --- | --- | --- | --- | --- | --- | --- |
| 1 | 2 | 3 | 4 | 5 | 6 | 7 |

**6.** Please rate how much the COVID-19 pandemic has impacted your **motivation to lose weight**. during the past 30 days. Scores can range from “much less motivated” (1) to “much more motivated” (7).

| Much less motivated | Less motivated | Slightly less motivated | No change | Slightly more motivated | More motivated | Much more motivated |
| --- | --- | --- | --- | --- | --- | --- |
| 1 | 2 | 3 | 4 | 5 | 6 | 7 |

**7.** Please tell us the biggest challenge related the COVID-19 pandemic you have experienced in progressing toward your weight loss goals?

**8.** Is there anything else you would like to share with our team regarding how the COVID-19 pandemic has affected your participation in our study or path toward your weight loss goals?
