# Supplementary material for: Improving lifestyle obesity treatment during the COVID‐19 pandemic and beyond: New challenges for weight management
Source: Obes Sci Pract. 2021 Jul 1;8(1):32–44. doi: 10.1002/osp4.540 (PMC8441901; doi:10.1002/osp4.540)
Supplement: Supplementary file 2 — Supplementary Material [file OSP4-8-32-s001.docx]

**TITLE:** Improving Lifestyle Obesity Treatment During the COVID-19 Pandemic and Beyond: New Challenges for Weight Management

**AUTHORS:** Ann E. Caldwell, PhD^1,2^, Elizabeth A. Thomas MD^1,2,3^, Corey Rynders PhD^4,5^, Brooke Dorsey Holliman, PhD^6,7^, Cathryn Perreira, MA^7^, Danielle M. Ostendorf, PhD^1,2^, Victoria A. Catenacci MD^1,2^

**AFFILIATIONS:**

^1^ Department of Medicine, Anschutz Health and Wellness Center, University of Colorado Anschutz Medical Campus, Aurora, CO, USA

^2^ Division of Endocrinology, Metabolism, and Diabetes, Department of Medicine, University of Colorado Anschutz Medical Campus, Aurora, CO, USA

^3^Rocky Mountain Regional Veterans Affairs Medical Center, Aurora, CO, USA

^4^ Division of Geriatric Medicine, Department of Medicine, University of Colorado Anschutz Medical Campus, Aurora, CO, USA

^5^Eastern Colorado Veterans Affairs Geriatric Research, Education, and Clinical Center, Denver, CO, USA

^6^Department of Family Medicine, University of Colorado Anschutz Medical Campus, Aurora, CO, USA

^7^Adult and Child Consortium for Health Outcomes Research and Delivery Science (ACCORDS), Children’s Hospital Colorado, University of Colorado Anschutz Medical Campus, Aurora, CO, USA

**Supplementary Materials 2**. *Details on qualitative data analysis methodology*.

Content analysis uses characteristics and content of language to provide understanding of qualitative data through systematic classification, coding, and theme identification (10). The questions were answered by three groups of participants (cohorts 2 and 3 of DRIFT-2 and cohort 3 of TRE). Responses were divided by question and then by group, resulting in six sets of responses (three sets of responses for each question). These responses were then uploaded to Atlas.ti (Version 8) for data management. The analysis team (BDH and CP) independently reviewed a subset of data to inductively identify a set of codes. The research team met to review their respective code lists, reconciling any discrepancies in the code definitions and applications. This process continued until a final codebook was established, at which point the research team applied the final codes to the remainder of the transcripts and continued to meet regularly to resolve any coding discrepancies through consensus. To establish confirmability, 20% of the data were coded by both team members. Codes were then clustered into related categories which guided theme development and considered code frequency across all responses. Coded data were analyzed within and across the different study types.

A total of 122 responses were collected and analyzed from the open-ended questions regarding 1) the biggest challenge participants experienced in relation to the pandemic and their weight loss goals (77 responses), and 2) anything else the participant wanted to share with study staff about how COVID-19 impacted their participation in the study and path toward their goals (45 responses). Each cohort’s responses were examined individually and then compared. No significant thematic differences were found between the three groups of respondents.
